# The Effect of Dietary Components of the Mediterranean Diet on Food Allergies: A Systematic Review

**DOI:** 10.3390/nu15153295

**Published:** 2023-07-25

**Authors:** Eleftheria Panagiotou, Eleni Andreou, Stella A. Nicolaou

**Affiliations:** Department of Life Sciences, School of Life and Health Sciences, University of Nicosia, 2417 Nicosia, Cyprus; panagiotou.e1@live.unic.ac.cy (E.P.); andreou.el@unic.ac.cy (E.A.)

**Keywords:** food allergies, Mediterranean diet, MD, olive oil, polyphenols, long-chain omega-3 fatty acids

## Abstract

Allergies are a common and increasing health problem affecting millions of people worldwide. This increase is attributed to genetic predisposition, air pollution, climate change, lack of physical activity, and alterations in eating habits. The Mediterranean diet (MD), which includes a lot of fruits and vegetables, whole grains, legumes, nuts, olive oil, and fish, has been linked to a variety of health benefits, including a lower risk of chronic and allergic disease. This paper explores the effects of the dietary components of the MD on food allergies. Electronic databases PubMed, Scopus, Science Direct, and EBSCO were used to conduct this systematic review. Out of 696 studies initially identified, five human and four animal studies were included. Risk of bias was determined using the Office of Health Assessment and Translation tool. In human studies, when the intervention was given during pregnancy and lactation, a beneficial effect was observed. When the intervention was given during pregnancy and until birth or to the infant for six months, no effect was observed. The animal studies indicated a beneficial effect between the food components of the MD and food allergies. Although the results are promising, the limited number of studies highlights the need for more research.

## 1. Introduction

Allergies are a growing global public health problem, and it is estimated that food allergies affect up to 10% of the global population [1,2]. Asthma, allergic rhinitis, seasonal eczema, dermatitis, atopy, and food allergies are, in general, the most common allergies that people develop [3]. Allergies are characterized by abnormal adaptive immune responses that may or may not involve allergen-specific IgE. During an allergic inflammatory response, sensory nerves become sensitized and activated, causing a variety of symptoms [4]. Severity of allergic reactions varies from person to person and can range from a minor irritant reaction to anaphylaxis, a potentially life-threatening emergency. Some of the allergy symptoms that may occur include itching, swelling, shortness of breath, vertigo, and loss of consciousness [2,3].

Food allergy diagnostic procedures include skin prick testing, food-specific IgE measurement, and elimination diets, whilst the golden standard is a double-blind, placebo-controlled food challenge [5]. Assuming that certain foods are suspected to be the cause of an allergic disorder, they can be eliminated from the patient’s diet to alleviate symptoms and confirm the diagnosis. The Food Standard Agency identifies 14 different foods as the most common causes of allergic reactions. These include celery, gluten-containing cereals (wheat, rye, barley, and oats), crustaceans, eggs, fish, lupin, milk, mollusks, mustard, peanuts, sesame, soybeans, sulfur dioxide and sulfites, as well as tree nuts (cashew nuts, almonds, and hazelnuts) [6].

In addition to genetic predisposition, this increase in allergic cases is likely to be attributed to air pollution, climate change, more time spent indoors, lack of physical activity, and alterations in people’s eating habits, among others [7,8]. In today’s modern world, people eat more foods that have been processed, altered, and modified; they consume fewer fruits and vegetables; and they consume excessive amounts of sugar, saturated fat, and junk food [9,10].

In the last few years, people have turned to healthier diets such as the Mediterranean diet (MD). Olive oil, as the primary source of fat, is the most vital component of the MD. MD is characterized by high consumption of whole grains, vegetables, beans, fruits, nuts, and seeds, and moderate amounts of fish, seafood, dairy products, and poultry. In contrast, red meat, sweets, sugary drinks, and butter are consumed rarely [8,10,11,12]. The MD is high in antioxidants, carbohydrates, and fiber and low in saturated fatty acids. It also contains a high concentration of monounsaturated fatty acids and *n*-3 polyunsaturated fatty acids (PUFAs) [9] and is rich in polyphenols, naturally occurring compounds present in a wide range of plant-based foods such as fruits, vegetables, nuts, and seeds. The MD provides various health benefits, including a lower risk of cardiovascular disease and metabolic syndrome, as well as promoting a healthy balance of gut microbiota in the digestive tract. It assists individuals in maintaining normal levels of glucose, blood pressure, and cholesterol.

Recent evidence also suggests that people with allergies may benefit from the MD. This may occur due to the high levels of antioxidants (such as vitamins, minerals, and fatty acids) and anti-inflammatory substances found in the MD that boost immune function [9]. Kontogianni’s (2008) study investigated the rates of MD adherence in a sample of Greek children and adolescents. Even though MD consumption by children appears to have a protective effect against asthma/wheezing, the findings demonstrate low adherence rates [9,13]. The role of the MD in allergic disease has been previously discussed in two comprehensive systematic reviews by Castro-Rondriguez et al. (2017) and more recently by Koumpagioti et al. (2022) [9,10]. According to the findings of both systematic reviews, children’s adherence to the MD appears to have a protective effect on asthma but not on allergic rhinitis, eczema, or atopy symptoms [9,10]. Also, both studies highlight the need for further research due to the studies’ remarkable heterogeneity [9,10]. Furthermore, a systematic review by Garcia-Marcos (2013) aimed to evaluate the relationship between adherence to the MD and the risk of developing asthma in children. The results showed that greater adherence to the MD was associated with a lower risk of developing asthma in children [12].

To the best of our knowledge, there is no systematic review addressing the association of the MD or its nutritional components with the development of food allergies. As discussed above, the MD is rich in polyphenols and polyunsaturated fatty acids (PUFAs), particularly omega-3 fatty acids, and evidence suggests that these compounds may play a role in lowering the risk of developing food allergies [14]. As such, the purpose of the current systematic review is to determine any association between the food components of the MD and food allergies.

## 2. Materials and Methods

### 2.1. Study Design

The current review followed the PRISMA 2020 statement for systematic reviews [15]. The review protocol has been registered in PROSPERO—International prospective register of systematic reviews (ID: CRD42023408827). The systematic literature review was performed using the electronic databases PubMed, Scopus, ScienceDirect, and EBSCO. The following keywords and their combinations were used with Boolean logic: (“food allergies”) AND ((“olive oil”) OR (polyphenols) OR (long-chain omega-3 fatty acids)). Only published peer-reviewed studies up to March 1st 2023 were included. There were no restrictions on language. Search and study selection were performed by three independent reviewers (E.P, S.A.N., and E.A.).

### 2.2. Inclusion and Exclusion Criteria

The PICOS (Population, Intervention, Comparison/Comparator, Outcomes, Study Type) methodology was utilized to select all eligible studies. The inclusion criteria for this review included (i) participants of any age (e.g., infants and pregnant women and their infants) or food allergy animal models; (ii) cohort, cross-sectional, randomized controlled trials, observational studies, and experimental studies; (iii) studies that state the effect of a food component of the MD (e.g., polyphenols, olive oil, long-chain omega-3 fatty acids on food allergies); and (iv) studies published from 2000 until March 2023. Studies were excluded if (i) they did not follow the inclusion criteria or (ii) if they investigated other diets or (iii) if they investigated other allergies/diseases.

### 2.3. Study Selection

Following the database search, duplicates were identified and removed. Next, the titles and abstracts of all publications were screened for relevance according to the inclusion and exclusion criteria. All irrelevant studies were removed. If the result was not obviously irrelevant, the entire document was downloaded and reviewed.

### 2.4. Data Extraction

For human studies, data extracted included information on the author, year, sample size, country, study design, food component, methodology, and results (effect of the food components of the MD on allergy symptoms).

For animal studies, data extracted included information on author/year, # of animals, age, strain/species, sex, component/dose, route, sensitization day, challenge day, and results.

### 2.5. Risk of Bias for Included Studies

To assess the risk of bias, the Office of Health Assessment and Translation (OHAT) tool was used [16]. For the animal studies, 9 domains were included (risk in randomization, allocation, experimental conditions, blinding during the study, incomplete data, exposure characterization, outcome assessment, and reporting). For the human studies, the same bias domains were included except for the risk in experimental conditions. Each domain was marked as follows: (++) Definitely Low; (+) Probably Low; (-) Probably High; (--) Definitely High; and (NA) Not Applicable.

## 3. Results

### 3.1. Selection of Studies

**Figure 1:**
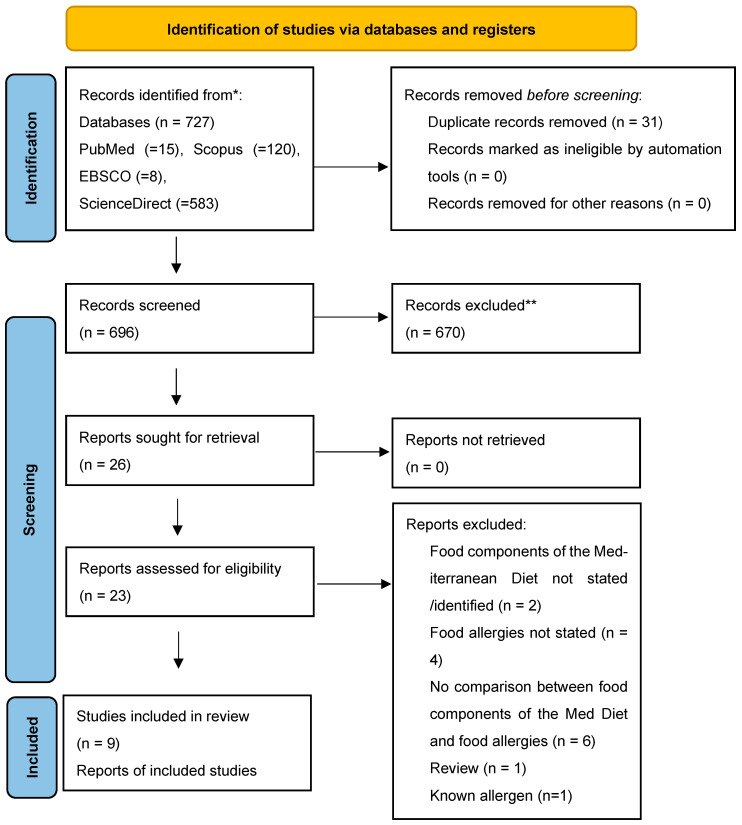
Schematic of selection of studies, PRISMA 2020 [15]. * Consider, if feasible to do so, reporting the number of records identified from each database or register searched (rather than the total number across all databases/registers). ** If automation tools were used, indicate how many records were excluded by a human and how many were excluded by automation tools.

From the 727 identified articles by the literature search, 31 were duplicates and 670 did not meet the criteria for inclusion, so they were excluded. Of the 23 articles evaluated for eligibility during the full-text screening, 12 failed to meet inclusion criteria. In the end, nine articles met the inclusion criteria for this systematic review, as shown in Figure 1.

### 3.2. Quality Assessment

The OHAT tool was used to evaluate the risk of bias in this systematic review (Table 1) [16]. The tool enables us to check both human and animal studies via excluding/including specific domains. All studies included in the current review were of high quality.

### 3.3. Study Characteristics

This study included a total of nine studies, of which five were conducted on humans (randomized controlled trial, *n* = 4; cross-sectional, *n* = 1) and four were conducted on animals (experimental, *n* = 4). The human studies were conducted in Sweden (*n* = 2), Australia (*n* = 2), and China (*n* = 1), while the animal studies were conducted in China (*n* = 2) and Switzerland (*n* = 2).

In two of the human studies by Furuhjem et al. (2009, 2011), researchers followed pregnant mothers at risk of having an allergic infant (*n* = 290) from the 25th week of pregnancy and their infants for either 12 or 24 months [17,19]. In these two studies mothers received LCPUFA supplementation from the 25th week of gestation and for 3–4 months of lactation. Palmer et al. (2012) followed pregnant mothers at risk of having an allergic infant (*n* = 706) from the 21st week of pregnancy and their infants for 12 months [14]. In this study mothers, received LCPUFA until birth. In the study by D’Vaz et al. (2015), infants (birth to six months of age) received fish oil supplementation, and the effect on allergic disease was investigated [18]. The fifth human study conjugated the known cow milk allergen bovine β-lactoglobulin (βLG) to the polyphenols epigallo-catechin 3-gallate (EGCG) and chlorogenic acid (CA) to reduce the allergenicity of cow’s milk [20].

All four animal studies used the ovalbumine (OVA)-sensitized BALB/c mouse model as their food allergy model. The two studies by Ma et al. (2022, 2023) fed their animals olive oil prior to sensitization. In the studies by Singh et al. and Zuercher et al. [23,24], they fed their mice with enriched polyphenols (either epicatechin or flavonol). Table 2 and Table 3 summarize the characteristics of the human and animal studies, respectively.

### 3.4. Effect of Components of the MD on Food Allergy in Infants and Animal Models

Four of the human studies investigated the effect of PUFAs on food allergies [14,17,18,19]. Fujuhjem (2009 and 2011) initiated the supplementation during pregnancy and the first month of lactation and found a beneficial effect, while Palmer (2012) and D’Vaz (2012) supplemented either the mother until birth or the infant until six months and found no effect [14,17,18,19]. The fifth human study investigated the effect of conjugating an allergen with polyphenols and showed that IgE-binding capacity was lowered, indicating a beneficial effect [20].

Two of the animal studies investigated the effect of olive oil on food allergies and demonstrated a beneficial effect [21,22]. This was demonstrated by a does-dependent inhibition of allergen-specific antibodies (IgG and IgE) as well as a decrease in the expression of TGF-β [21]. The second study also found a decrease in clinical symptoms of allergy (dose-dependent) as well a a decrease in IgE, mouse mast cell protease (mMCP)-1, and TNF-α levels in the olive oil group (*p* < 0.01). The other two animal studies invesigated the effect of polyphenols on food allergies [23,24]. The study by Singh et al. (2014) used epicatechin and showed a dose-dependent decrease in clinical symptoms and IgE [24]. Similarly, Zuercher et al. (2010) used flavonol enriched polyphenols and showed a decrease in the severity of allergic symptoms, while IgE levels remained unchanged and cytokine data were inconclusive [23]. To determine clinical severity, allergic symptoms were graded (scratching, rubbing/swelling around snout and mouth, piloerection, wheezing, lack of activity, tremor/convulsions, and death).

**Table 2:** Summary of studies conducted with humans reporting any association between food allergies and food components of the MD.

Author/Year	Country	Duration	Sample Characteristics	Study Design	Food Component of Med Diet	Methodology	Results— Effect on Food Allergies Symptoms
**Furuhjelm et al., 2011** [17]	Sweden	24 month infant follow-up	145 pregnant women (25th week) at risk of having an allergic infant and their infants	Randomized controlled trial	Long-chain unsaturated fatty acids (LCPUFA): docosahexaenoic acid (DHA) and eicosapentaenoic acid (EPA) or placebo	Intervention group daily dose of: 1.6 g EPA and 1.1 g DHA or placebo from the 25th week of pregnancy to the first 3.5 months of breastfeeding Control group: placebo	(a) Dose-dependent beneficial effect in allergic sensitization and IgE-associated disease in the infant (*p* = 0.01–0.05) (b) No obvious preventive effect on symptoms of allergic disease
**Palmer et al., 2012** [14]	Australia	12 month infant follow-up	706 infants (Follow-up study of DHA to Optimize Mother Infant Outcome [DOMInO] trial)	Randomized controlled trial	Fish oil capsules (*n*-3 –LCPUFA; DHA and EPA)	Intervention group (*n* = 368): daily dose of fish oil capsules (900 mg of *n*-3 LCPUFA; 800 mg DHA and 100 mg EPA) from 21 weeks’ gestation until birth. Control group (*n* = 338): capsules of vegetable oil without *n*-3 LCPUFA	No effect in IgE associated food allergy Unadjusted relative risk: 0.68; 95% confidence interval: 0.43 to 1.05, *p* = 0.08; adjusted relative risk: 0.7, 0.45 to 1.09, *p* = 0.12
**D’Vaz et al., 2012** [18]	Australia	6 months	420 infants	Randomized controlled trial	Fish oil (DHA and EPA) or a control olive oil.	Intervention group (*n* = 218): From birth to 6 months, received a daily supplement of fish oil comprising 280 mg DHA and 110 mg EHA Control group (*n* = 202): olive oil.	No effect on allergic outcomes including food allergy
**Furuhjelm et al., 2009** [19]	Sweden	12-month infant follow-up	145 pregnant women (25th week) at risk of having an allergic infant and their infants	Randomized controlled trial	EHA and DHA or placebo	Intervention group (*n* = 52): daily dose, 1.6 g EHA and 1.1 g DHA from the 25th gestational week—3–4 months of breastfeeding. Control group (*n* = 65): soy oil capsules	Beneficial effect Compared to the placebo group (10/65, 15%), the omega-3 group had a reduced rate of food allergy (1/52, 2%; *p* < 0.05)
**Wu et al., 2018** [20]	China		Sera from 10 children with cow milk allergy.	Cross-sectional study	Bovine β-lactoglobulin (βLG) (cow milk allergen) conjugated with epigallo-catechin 3-gallate (EGCG) and chlorogenic acid (CA)	A pool of sera from children with cow milk allergy (*n* = 10) and a pool of sera from individuals without the allergy (*n* = 5) Were exposed βLG conjugated with EGCG and CA	Beneficial effect. βLG conjugated with polyphenols was effective in lowering IgE-binding capability (*p* < 0.05).

EPA: eicosapentaenoic acid; DHA: docosahexaenoic acid; LCPUFA: long-chain polyunsaturated fatty acids; βLG: bovine β-lactoglobulin, EGCG: epigallo-catechin 3-gallate; CA: chlorogenic acid.

**Table 3:** Summary of studies conducted with animals reporting any association between food allergies and the food components of the MD.

Author/Year	# Of Animals	Age	Strain/Species	Sex	Food Component of Med Diet	Sensitization Day	Challenge Day	Methodology	Results – Effect of Food Components of Med Diet on Food Allergies Symptoms
**Ma et al., 2022** [21]	48	4 weeks of age	BALB/c mice. Ovalbumine (OVA)-sensitized mice (FA model)	Female	Olive oil	14th day	33rd day	Experimental groups: 2 weeks of olive oil prior to sensitization. 1.0 or 2.0 or 3.0 g/kg·day olive oil with and without sensitization, phosphate-buffered saline (PBS) group and OVA group.	Beneficial effect. Dose-dependent inhibition of IgG, IgE, and histamine. Dose-dependent increase o TGF-β expression in the ilea of allergic mice.
**Ma et al., 2023** [22]	15	3–5 weeks of age	BALB/c mice. OVA-sensitized mice	Male	Olive oil	14th day	28–40th day	Experimental groups: 2 weeks of olive oil prior to sensitization to olive oil group, allergy model group, and PBS group.	Beneficial effect. Decrease in clinical symptoms of allergy. Decrease in IgE, mouse mast cell protease (mMCP)-1, and TNF-α levels in the olive oil group (*p* < 0.01). Positive effect on intestinal epithelial mucosal immunity.
**Zuercher et al., 2010** [23]	10–15/group	6 weeks old	BALB-c mice per group. OVA-sensitized mice	Female	Polyphenol enriched apple extract (flavonols)	1–42nd day	49th day	Experimental groups: OVA-sensitized mice fed with the apple extract at Day 0 (primary prevention) or Day 42 (secondary prevention, positive and negative control.	Beneficial effect. Decrease in the severity of allergic symptoms and mediator release (mMMCP-1) comparable in Day 0 and Day 42 groups. IgE level remained the same. Variable cytokine data.
**Singh et al., 2014** [24]	10–15/group	6 weeks old	BALB-c mice per group. OVA-sensitized mice	Female	Polyphenol-enriched apple extracts A and B, polyphenol-enriched with cocoa or polyphenol-enriched with epicatechin	3rd day	49th day	Experimental groups: OVA-sensitized mice fed with the extracts, cholera toxin-sensitized mice (negative control), and regulary fed mice (positive control).	Beneficial effect. Dose-dependent decrease in clinical symptoms of allergy and IgE in mice given the epicatechin extract (*p* = 0.01).

FA: food allergy; OVA: ovalbumin; PBS: phosphate-buffered saline; mMCP: mouse mast cell protease; TGF-β: transforming growth factor-beta; IgE: immunoglobulin E; mMCP-1: TNF-α: tumor necrosis factor-α.

## 4. Discussion

The aim of the current systematic review was to determine whether components of the MD such as olive oil, polyphenol, or PUFAs have any effect on food allergies in infants and animal models. The human studies indicated that when the intervention occurs during pregnancy and though lactation, a beneficial effect is observed [17,19]. However, when the intervention is given to pregnant mothers either until birth or to the infants for their first six months of life, no effect is observed [14,18]. All the animal studies indicated a positive relationship between the food components of the MD and food allergies. The food components used were olive oil and enriched polyphenols [21,22,23,24].

Epidemiological studies have demonstrated that the prevalence of food allergies in Western nations is correlated with the excessive consumption of *n*-6 PUFAs in the diet [2]. In the MD, *n*-3 PUFAs are predominant and more beneficial. According to the findings, two of the randomized controlled studies included in this review, ω-3 supplementation during pregnancy and breastfeeding, may lower the risk of food allergy during the first year of life [17,19]. Also, a cohort study by Chatzi et al. (2017) investigated the association between MD adherence during pregnancy and childhood respiratory outcomes [25]. The findings suggest that higher adherence to the MD during pregnancy is associated with a reduced risk of childhood wheezing and asthma; however, three other studies showed no effect [9,25]. Another study that examined the link between asthma, rhinoconjunctivitis, obesity, exercise, and the MD in children in Spain showed a lower risk of asthma and rhinoconjunctivitis [26].

One way of assessing allergic outcomes is food allergen-specific IgE. In the papers included herein, higher maternal and newborn proportions of docosahexaenoic acid (DHA) and eicosapentaenoic acid (EPA) were related to a decreased prevalence of IgE-associated illness (*p* = 0.01–0.05) in a dose-dependent manner [19]. According to the findings of a systematic review conducted by Sartorio (2021), supplementation with PUFAs inhibits allergic response in preclinical studies [27]. Still, other studies investigating general atopy indicated that long chain PUFA supplementation during pregnancy did not decrease the general rate of IgE-associated allergies in the first year of life; however, atopic eczema and egg sensitization were reduced [14,17]. This was also noted in two of the human studies discussed here, as PUFAs did not show any effect [14,18], which may due to timing and dosing effects. To illustrate, despite the relatively large dose of *n*-3 PUFA utilized in the D’vaz et al. research study, only a small increase in *n*-3 PUFA levels was observed, which may indicate problems with the bioavailability and absorption of supplements [14]. The results of clinical studies regarding the optimal timing and dosages of supplementation, as well as the individuals most likely to benefit, are still contradictory; consequently, definitive conclusions cannot yet be drawn [28].

An alternative to studying human disease is with animal models, and food allergy is no exception. There are a number of animal models that mimic the human clinical features of food allergy, and these are discussed elsewhere [29,30]. These food allergy models show a tendency for Th2 responses that are expected in allergic responses [29,30]. In the current study, the same animal model, with variations in sensitization, was used (OVA-sensitized BALB/c mice with adjuvant). The animals were fed either olive oil or polyphenols, which are key nutritional components of the MD.

Olive oil consumption has been linked to several health benefits. Olive oil has direct (tocopherols, polyphenols, and mono-unsaturated fatty acids) and indirect (lower saturated fats and balanced linoleic/alpha linolenic acid) effects on the immune system and inflammatory responses (lower saturated fats and balanced linoleic/alpha linolenic acid) [31]. Two of the experimental studies included in this review used a mouse model to investigate the relationship between olive oil and food allergies [21,22]. These two studies found that administering olive oil orally to mice improved epithelial mucosal immunity [21,22]. Further, olive oil alleviates the symptoms of OVA-induced anaphylaxis, lowers intestinal inflammation, and reduces the level of OVA-specific IgE and anaphylactic mediator in the serum [21]. Over 8000 distinct polyphenols are found in fruit, vegetable, and cereal. Recent studies have identified a protective role of polyphenols against food allergies and investigated the underlying mechanisms [32]. Also as shown in this review, polyphenols showed a beneficial effect on food allergies in mice [23,24]. Polyphenols can bind to allergens either covalently or noncovalently, forming conjugates or complexes. These changes cause secondary structural changes in the proteins (like less helical and sheet content), which covers the immunoglobulin E (IgE) epitope and changes the way antigens are presented, stopping the allergic reaction in vivo. Likewise, in the cellular model, the complexes inhibited the degranulation of rat basophil leukemia cells via the mitogen-activated protein kinase signaling pathway, resulting in a decrease in hexosaminidase and histamine levels [19,27,32].

The current study has some limitations. Initially, high-quality human studies were sought. However, the search indicated that there are a limited number of human studies that investigated the effect of the components of the MD on food allergies, and the search was expanded to include animal studies as well. As a result, there is heterogeneity in terms of research design, methodology, and characteristics. Still, this enables a more holistic view of the available studies. This review’s strength is that inclusion and exclusion criteria were clearly established and consistently applied across all studies. The current review highlights the need for more research in the field.

## 5. Conclusions

The findings of the current study point to an overall beneficial effect between the MD and the development of food allergies. This is not surprising as the MD is well known for its health-promoting and anti-inflammatory properties, as it contains valuable nutrients such as *n*-3 long chain PUFAs, polyphenols, and other fat-soluble micronutrients. Specifically, when the diet of pregnant women through lactation was supplemented with long chain PUFAs, a beneficial effect in the development of food allergies and allergy symptoms was observed in the infant [17,19]. This effect was lost if the supplementation was only given to pregnant women either until birth or to the infant for six months [14,18]. All of the animal studies indicated a positive relationship between the food components of the MD (olive oil and enriched polyphenols) and FAs [21,22,23,24].

At present, there is an increasing demand for processed foods in the global market [2]. Thus, understanding the basic and current information behind the function of food components in allergy is an urgent need. This may enhance diagnosis and treatment and point to prevention measures for food allergy. In support of this, the current review has demonstrated that *n*-3 long chain PUFAs and polyphenols, both components of the MD, appear to have a positive effect on food allergies. Further this study highlights the limited number of studies addressing this topic, indicating that more research on the field is warranted.

## Figures and Tables

**Table 1:** Risk of bias assessment of included studies using the OHAT Risk of Bias Rating Tool.

	Author/Year	D1	D2	D3	D4	D5	D6	D7	D8	D9	D10
Human	(Furuhjelm et al., 2011) [17]	++	++	NA	NA	NA	++	++	+	++	++
	(Palmer et al., 2012) [14]	++	++	NA	NA	NA	++	++	+	++	++
	(D’Vaz et al., 2012) [18]	++	++	NA	NA	NA	++	+	+	++	++
	(Furuhjelm et al., 2009) [19]	++	++	NA	NA	NA	++	+	+	++	++
	(Wu et al., 2018) [20]	NA	NA	++	--	NA	NA	++	+	+	+
Mice	(Ma et al., 2022) [21]	++	+	NA	NA	++	+	++	+	+	++
	(Ma et al., 2023) [22]	++	+	NA	NA	++	+	++	+	+	++
	(Zuercher et al., 2010) [23]	++	+	NA	NA	++	+	++	+	+	++
	(Singh et al., 2014) [24]	++	+	NA	NA	++	+	++	+	+	++

Scoring: (++), Definitely Low, shown in light grey; (+), Probably Low, shown in dark grey; (--), Definitely High, shown in very dark grey; NA, Not Applicable. Domains Judgement: D1: Risk in randomization; D2: Risk in allocation concealment; D3: Risk in comparison groups; D4: Risk in confounding factors; D5: Risk in experimental conditions; D6: Risk in blinding during study; D7: Risk due to incomplete data; D8: Risk in exposure characterization; D9: Risk in outcome assessment; D10: Risk in reporting.

## Data Availability

Not applicable.
